# Role of rapid antigen detection test for the diagnosis of group A beta-hemolytic streptococcus in patients with pharyngotonsillitis

**DOI:** 10.1016/S1808-8694(15)31306-9

**Published:** 2015-10-20

**Authors:** Bernardo Cunha Araujo Filho, Rui Imamura, Luiz Ubirajara Sennes, Flávio Akira Sakae

**Affiliations:** ^1^ Otorhinolaryngologist (residence program, HCFMUSP), Specialist in ORL, SBORL (Ph.D. studies under course, Division of Clinical Otorhinolaryngology, HCFMUSP); ^2^ Assistant Physician, Discipline of Otorhinolaryngology, Medical School, University of Sao Paulo (Assistant Physician, Ph.D., Discipline of Otorhinolaryngology, Medical School, University of Sao Paulo); ^3^ Full Professor, Discipline of Otorhinolaryngology, Medical School, University of Sao Paulo (Full Professor, Discipline of Otorhinolaryngology, Medical School, University of Sao Paulo); ^4^ Otorhinolaryngologist (residence program, HCFMUSP), Specialist in Otorhinolaryngology, SBORL (Ph.D. studies under course, Division of Clinical Otorhinolaryngology, Hospital das Clínicas, Medical School, University of Sao Paulo). Study conducted at the Division of Clinical Otorhinolaryngology, Hospital das Clínicas, Medical School, University of Sao Paulo

**Keywords:** rapid antigen detection test, group A pyogenes streptococcus, pharyngotonsillitis

## Abstract

Group A *β* -hemolytic streptococcus (GAS) is an important pharyngotonsillitis etiologic agent. Correct etiologic diagnosis and early treatment prevent suppurative and non-suppurative complications of streptococcal pharyngotonsillitis; however, clinical diagnosis is not reliable. Within this context, rapid detection methods of GAS antigen are useful to diagnose this agent.

**Aim:**

The objective of the present study was to determine sensitivity and specificity of rapid GAS antigen detection tests used in Brazil.

**Study design:**

Clinical prospective. **Method**: Eighty-one patients with clinical diagnosis of acute pharyngotonsillitis seen at the otorhinolaryngology emergency department of the University Hospital, FMUSP, between May 2001 and April 2002 were submitted to two simultaneous collections of oropharyngeal material using swabs. The rapid GAS antigen detection test was compared to culture on blood agar, the gold standard for the diagnosis of this etiologic agent.

**Results:**

Among the 81 patients studied, the rapid test was positive in 56% and negative in 44%. GAS growth in culture was observed in 40.7% of the patients. Sensitivity and specificity of the rapid test were, respectively, 93.9% and 68.7%, and the negative and positive predictive values were 94.2 and 67.4%, respectively. **Conclusions**: We concluded that high sensitivity of the test allows its use in the identification of patients with GAS. Rapid streptococcal antigen detection tests have been shown to be an important adjuvant tool in the etiologic diagnosis of pharyngotonsillitis.

## INTRODUCTION

Pharyngotonsillitis (PT) caused by group A *β -hemolytic Streptococcus* (GAS) is a common affection in Brazil, affecting mainly children and young adults. GAS infection is the main bacterial etiology of acute PT, affecting 15 to 30% of all cases in children and adolescents and 5 to 10% of the cases in adults^1^, ^2^, ^3^.

PT may be caused by viral or bacterial infections, but in rare exceptions (*Corynebacterium diphtheriae* and *Neisseria gonorrhoeae*) only infections caused by GAS have formal indication of treatment with antibiotics^4^, ^5^. In such cases, treatment with antibiotics shortens symptoms related with infection (if used within 48 hours after onset of symptoms), it may avoid suppurative and non-suppurative complications and prevent dissemination to the community^2^, ^6^, ^7^, ^8^, ^9^, ^10^. Rheumatic fever and glomerulonephritis, followed by suppurative complications (abscesses, bacteremia, endocarditis) are the most feared complications^11^.

Given that most acute PT are caused by other agents, virus for example, they do not require treatment with antibiotics. It is extremely important for clinicians and otorhinolaryngologists to be capable of ruling out streptococcus PT, preventing inappropriate use of antibiotics in non-streptococcus PT, exposing patients to unnecessary expenses and the inherit risks of antibiotic use, in addition to increased bacterial resistance^7^, ^9^.

In the USA, 70% of PT are treated with antibiotics^4^, ^5^, and it is believed that in Brazil there is higher volume of PT treated this way.

As a result of variability of clinical presentations of streptococcus PT and the large number of agents capable of producing similar clinical presentation, clinical diagnosis of PT caused by GAS is not always reliable^10^, ^12^. Thus, the physician has to make use of some laboratory methods in an attempt to conclude etiological diagnosis. In the 80's, the market received some rapid antigen detection tests for GAS to have it diagnosed within few minutes. They are easy to handle and interpret tests, which can be used in medical offices^7^, ^8^. Thus, rapid test may support the etiological diagnosis and treatment of streptococcus pharyngotonsillitis. New techniques for rapid GAS antigen detection have been developed to make them more sensitive, cheaper and easier to use^10^; however, the experience of its use in public healthcare centers, in which it takes greater importance owing to social -economic reasons, is still limited.

The authors aimed at assessing sensitivity and specificity of rapid GAS antigen detection tests comparing them to the results of culture to define the role they have on daily practice of Otorhinolaryngologists.

## MATERIAL AND METHODS

We prospectively studied 81 patients with clinical diagnosis of PT seen in the Emergency Room of Otorhinolaryngology, Hospital das Clínicas, Medical School, University of Sao Paulo, between May 2001 and April 2002, who were selected to the study based on inclusion and exclusion criteria (Chart 1). Fifty-five (67%) were men and sixty-six (33%) were women. Mean age was 39.4 years, ranging from 18 to 69 years.

**Chart 1:** Criteria for inclusion and exclusion in the study.

**Inclusion**: 1. Men and women aged 18 or older. 2. Patients with clinical diagnosis of pharyngotonsillitis evidenced by: Presence of at least two symptoms: • Sore throat • Odynophagia • History of fever Presence of at least two signs: • Erythema and pharynx or tonsil edema • Uvular edema • Pharyngotonsillar exudate • Painful anterior neck ganglions • Temperature > 38º C • Leukocytes > 12000/mm
**Exclusion**: 1. Presence of the following symptoms: • Cough • Acute rhinorrhea 2. History of treatment with antibiotics for the past two weeks

For all patients, we collected two simultaneous swabs (Culturette; Becton Dickinson, Maryland) from the posterior wall of the oropharynx and tonsils by the same team of Otorhinolaryngologists. The first swab was taken to the laboratory and it was inoculated in sheep agar-blood plates at 5% for 24-48 hours, following conventional biochemical methods and confirmed by identification of automated system Untek Systems (Biomeniux, USA). The other swab was tested with rapid test, using the optical immune system Strep A OIA Max, within 5 minutes, according to manufacturers' instructions (International Microbio, USA). The laboratory technician did not know the results of rapid test. Any growth of *Streptococcus pyogenes* on the culture plate was considered a positive culture.

The results of the rapid test were compared with the results of the culture and sensitivity and specificity of the kit Strep A OIA Max were determined; laboratory culture was considered the gold standard method.

## RESULTS

A total of 81 oropharynx swabs were analyzed. Out of the total swabs, 46 (56%) were positive for rapid test and 35 (44%) were negative. Thirty-three (40.7%) of the patients had positive culture for *Streptococcus pyogenes* (Table 1). There were 2 false-negative results. Sensitivity of the rapid test was 93.9% (31 out of 33), specificity was 68.7% (33 out of 48), positive predictive value was 67.4% (31 out of 46) and negative predictive value was 94.2% (33 out of 35). Out of 5 positive cultures for other groups of *Streptococcus ²-hemolytic*, only one (group C *Streptococcus*) presented positive rapid test.

**Table 1:** Comparison of rapid test with culture for the detection of GAS antigen in 81 patients with pharyngotonsillitis.

METHOD	CULTURE		Total
	Positive	Negative	
Rapid Test IOA			
positive	31	15	46
negative	02	33	35
Total	33	48	81

Forty-five patients (55.5%) presented culture with presence of other bacteria.

## DISCUSSION

Similarly to Mayes et al.^7^, we used swabs for collection of sample of oropharynx bacteria obtaining positive results in almost all cultures. Out of 81 patients included in the study, 78 (96.2%) had bacterial growth in the culture, and 40.7% were positive for GAS, leading us to believe that the inclusion and exclusion criteria adopted were appropriate and indicative of bacterial pharyngotonsillitis, despite the fact that it could also correspond to healthy subjects and/or false-positives. In patients with negative culture for GAS, there was higher prevalence of saprophyte oropharyngeal bacteria.

We decided to use agar-blood culture medium in aerobe environment considering that studies developed comparing different culture methods showed that inoculation in this medium is as good as other more selective methods^8^, ^9^, ^13^. Agar-blood culture is the exam of preference for diagnosis of GAS, with sensitivity of 90 to 95%^6^, ^7^, ^12^. False-negative cultures are probably results of patients with small number of colonies, and many are holders. However, this exam may delay the recognition of streptococcus PT in 48 to 72 hours, preventing early diagnosis and antibiotic therapy would lose its value to abbreviate the symptoms of PT and reduce transmissions of GAS to other subjects^14^.

The prevalence of Streptococcus pyogenes in the studied literature comprised about 25% of all episodes of acute pharyngotonsillitis^7^, ^8^, ^9^, ^10^. According to Bisno et al., 5% to 10% of acute PT in adults have GAS as pathological agents, but we found 40.7% in our sample, which surprised us for two reasons: first because the prevalence of this agent in developing countries such as Brazil is markedly higher, owing to factors related with poor basic sanitation and deficient healthcare systems^10^, and secondly because the inclusion and exclusion criteria were selective for GAS.

The criteria were used in an attempt to select streptococcus PT, based on the criteria proposed by Centor (pharyngotonsillitis exudate, history of fever, neck lymphoadenopathy, and absence of cough). According to some authors, Centor criteria are the most reliable predictive clinical factors for diagnosis of PT caused by SGA^4^. It presented positive predictive value of 56% when meeting the four criteria, especially when applied in regions where the prevalence is high^4^, ^5^, which was confirmed in our study. However, the use only of clinical criteria for diagnosis would lead to unnecessary treatment in many patients.

Kit Strep OIA Max proved to be quick, practical and easy to use and interpret.

Sensitivity of rapid detection test in the literature ranged from 77% to 97%^8^. The great variability of sensitivity demonstrated by different authors may be resultant from some biases, such as knowledge of rapid test result during colony count of the culture, the selected culture methods, and inexperience of technicians^8^, ^10^. We found high sensitivity (93.9%), but less than proposed by Kellogg and Mozella^13^.

Specificity of 68.7% proved to be compatible with that of other studies, which related variability of 54% to 100%^9^. The increase in sample size could have contributed to improve this parameter. According to Hendley et al., studies sponsored by manufacturers of rapid test produced better results^8^. Similarly to that author's, our study was not sponsored by any company.

Pichichero et al. obtained only 2.4% of false-negative results, suggesting that rapid test would replace the culture for the diagnosis of infection by GAS^7^. The presence of two false-positive results (6%) in our study may have resulted from the small amount of antigen in the oropharynx. Even though the tests were negative, we observed slow bacterial growth in the culture. These cases can be explained by the low number of colonies that are detected only by culture^8^, ^10^. It may also occur in GAS holders without clinical disease, or when there is antigen block by anti-streptococcus antibody in the organism^13^. Moreover, inappropriate collection of material can also result in false-negative results^8^, ^13^.

False-positive results are more frequent, presented in 15 cases (32.6%), probably owing to rapid test method failure^8^, detecting non-specific bacterial antigens or owing to cross reaction with other groups of Streptococcus, such as in one of the cases (group C *Streptococcus*). Use of oral antiseptics may also generate false-positive results, because they prevent appropriate growth of organisms in the culture medium^4^, ^8^, but this possibility was not assessed in our study.

Negative predictive value of rapid test was 94.2% and it draws our attention to the fact that 6% of the negative tests were positive cultures. However, this is a small fraction of the population and complications would be rare, especially among adults, in the studied population, in which development of rheumatic fever is exceptional. This great sensitivity of rapid test would prevent patients from being submitted to culture, being treated with antibiotics based on positive test. Conversely, there are authors that advocate the conduction of culture in all patients with negative rapid test to minimize the risk of complications^7^, ^12^.

CDC (Centers for Disease Control and Prevention) in USA recommends treatment of all cases of PT by GAS. Cooper et al. suggested that in USA cultures should not be recommended as initial assessment method for patients with PT or for confirmation of negative results in rapid tests, when they clearly have sensitivity higher than 80%^4^. The need to assess sensitivity of the test in the Brazilian population and the unique profile of streptococcus PT in Brazil motivated the conduction of the present study.

We understand that the rapid test with negative result, considering it sensitivity, does not require further culture or treatment with antibiotics, once it is a very reliable method. In positive tests, it is worthwhile to manage the case with antibiotics. However, positive predictive value of 67.4% demonstrated that approximately 30% of the cases would be treated unnecessarily. Given the fact that we have high prevalence of GAS in our country, this is an extremely favorable result to the use of rapid GAS detection kit in Brazil. There is currently no study on cost-benefit ratio of the use of rapid tests. However, in the USA, despite the high cost of rapid test compared to culture, there are significant savings in prevention of indiscriminate use of antibiotics and additional visits to the doctor owing to side effects of these antibiotics^7^, ^9^.

## CLOSING REMARKS

*Streptococcus pyogenes* is the most prevalent organism in bacterial pharyngotonsillitis in our country.

Rapid streptococcus antigen test proved to be an important tool in diagnosis of pharyngotonsillitis owing to its high sensitivity. Thus, negative results point towards absence of streptococcus PT to be treated symptomatically, whereas positive results point towards diagnosis of streptococcus PT, which require antibiotics for its treatment.

We believe that new studies shall be conducted to determine cost-benefit ratio of these kits for detection and their impact on savings of healthcare services.
